# Tunable magnetic nanowires for biomedical and harsh environment applications

**DOI:** 10.1038/srep24189

**Published:** 2016-04-13

**Authors:** Yurii P. Ivanov, Ahmed Alfadhel, Mohammed Alnassar, Jose E. Perez, Manuel Vazquez, Andrey Chuvilin, Jürgen Kosel

**Affiliations:** 1 Computer, Electrical and Mathematical Sciences and Engineering Division (CEMSE), King Abdullah University of Science and Technology (KAUST), Thuwal, 23955, Saudi Arabia; 2 Biological and Environmental Sciences and Engineering Division (BESE), King Abdullah University of Science and Technology (KAUST), Thuwal, 23955, Saudi Arabia; 3 Institute of Materials Science of Madrid, CSIC, 28049 Madrid, Spain; 4 CIC nanoGUNE Consolider, Av. de Tolosa 76, 20018, San Sebastian, Spain; 5 IKERBASQUE, Basque Foundation for Science, Maria Diaz de Haro 3, 48013, Bilbao, Spain

## Abstract

We have synthesized nanowires with an iron core and an iron oxide (magnetite) shell by a facile low-cost fabrication process. The magnetic properties of the nanowires can be tuned by changing shell thicknesses to yield remarkable new properties and multi-functionality. A multi-domain state at remanence can be obtained, which is an attractive feature for biomedical applications, where a low remanence is desirable. The nanowires can also be encoded with different remanence values. Notably, the oxidation process of single-crystal iron nanowires halts at a shell thickness of 10 nm. The oxide shell of these nanowires acts as a passivation layer, retaining the magnetic properties of the iron core even during high-temperature operations. This property renders these core-shell nanowires attractive materials for application to harsh environments. A cell viability study reveals a high degree of biocompatibility of the core-shell nanowires.

In the past few years, various methods for the synthesis of different types of inorganic nanowires (NWs) have been developed^1^,^2^,^3^. Magnetic NWs in particular are attractive for a variety of technological applications ranging from flow sensors over bioinspired tactile sensors to cancer treatment^4^,^5^,^6^,^7^,^8^,^9^,^10^,^11^,^12^,^13^. Core-shell NWs comprising different core and shell materials can offer the advantage of combined properties from more than one material to convey multi-functional capabilities^14^,^15^. Tailoring the magnetic properties was successfully achieved in various sensor applications in core-shell metallic micro and nanowires^16^,^17^. Hybrid metallic/non-metallic nanowires offer specific advantages. For example, combining the high magnetization value of iron (Fe) with the biocompatibility and stability of iron oxide (Fe_3_O_4_) has this potential^18^,^19^,^20^,^21^.

Fe NWs have a high magnetic moment, and due to their single domain structure (a consequence of the large shape anisotropy), they exhibit relatively high coercivity and remanence magnetization values^22^. While a high remanence value can be an advantage when large forces are required, for example in cell separation applications^23^, it can also cause problems when magnetostatic interactions between them results in their agglomeration^20^. The high coercivity of NWs that increases when they are not aligned with the magnetic field^24^ prevents them from magnetization switching at low fields. This can be disadvantageous when magnetization losses are desired (e.g., in hyperthermia applications)^25^. Fe NWs oxidize relatively quickly, depending on the environmental conditions, leading to iron oxide NWs with much poorer magnetic moment^5^. Iron oxide has proven to be a viable material for biomedical applications in the form of superparamagnetic beads^26^,^27^,^28^,^29^,^30^,^31^,^32^. Hence, combining Fe and Fe_3_O_4_ to form core-shell NWs could present an attractive opportunity to tune their magnetic properties toward achieving long-term stability and a high degree of biocompatibility.

Core-shell NWs with Ni or Co cores and Fe_3_O_4_ shells have been previously reported, although their synthesis required expensive and complex processes. For instance, the walls of a porous aluminum oxide membrane were covered by atomic layer deposition of Fe_2_O_3_ followed by pore filling with Ni by electrodeposition and then H_2_ reduction to obtain the Fe_3_O_4_ shell^21^. Meanwhile, preparation of Co-Fe_3_O_4_ core-shell NWs required the use of a supercritical-fluid inclusion process in a porous aluminum oxide membrane^33^. In ref. 34. the authors studied the oxidation of the 200-nm diameter Fe wires embedded inside an aluminum oxide membrane. To date, however, the magnetic properties of individual magnetic core-magnetite shell NWs have no yet been reported.

In this work, we propose a process to synthesize Fe-Fe_3_O_4_ core-shell NWs with controllable geometric (core radius, shell thickness, and length) and crystalline (single or poly crystalline core) parameters. We introduce the magnetic properties of individual core-shell NWs to reveal new multifunctional and tunable properties.

## Results

**Figure 1:**
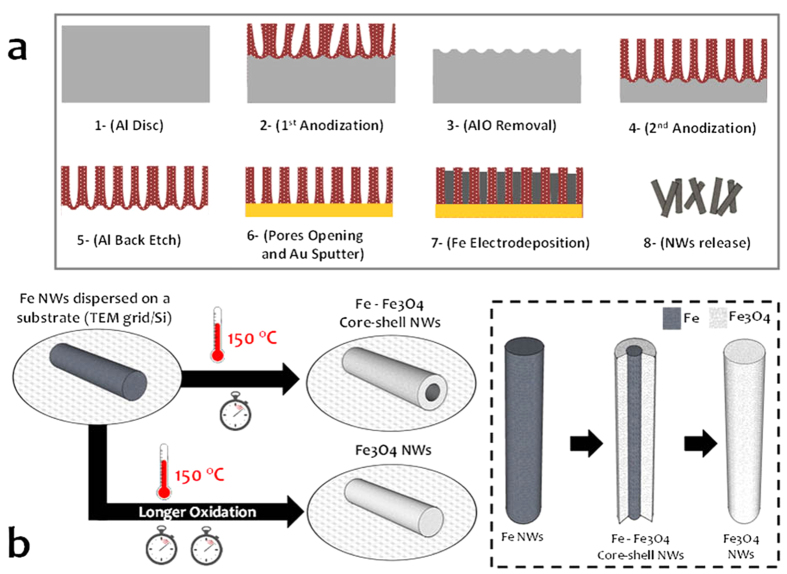
Fabrication process of the core-shell Fe-Fe_3_O_4_ nanowires (NWs). (**a**) Aluminum oxide template fabrication and Fe NW electrodeposition. (**b**) Formation of the core-shell structures.

The facile fabrication process consists of Fe NW electrodeposition onto aluminum oxide templates (AOT)^35^, the release of the NWs from the AOT and their subsequent oxidization in an oven (see Fig. 1). By controlling the electrodeposition conditions, we were able to synthesize either 15-μm long polycrystalline or 1-μm single-crystalline Fe NWs with a 50-nm diameter (see Methods). NW oxidation was performed in an oven at 150 °C between 10 minutes and 72 hours in an ambient atmosphere. The Fe_3_O_4_ shell was formed by heat-assisted oxygen diffusion between the grain boundaries to act as a passivation layer that prevents further oxidation of the bulk Fe core in regular conditions (e.g., room temperature).

**Figure 2:**
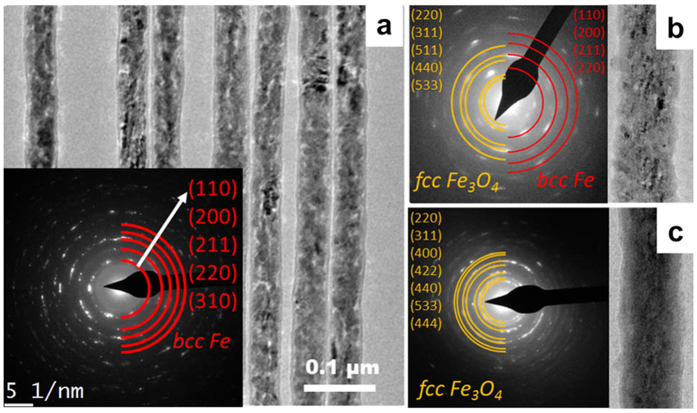
(**a**) Bright field TEM image and SAED pattern of polycrystalline Fe NWs before annealing. Fe-Fe_3_O_4_ core-shell NWs (**b**) after 20 min of annealing and (**c**) after 24 hours of annealing.

We used transmission electron microscopy (TEM) to evaluate the evolution of the core-shell NW structure for different oxidation times. Figure 2a shows the TEM image of several polycrystalline Fe NWs ranging in grain size from several nanometers to tens of nanometers. The rings found in the selected area electron diffraction (SAED) pattern, shown in the inset, identify the body-centered cubic (*bcc*) phase of Fe. An increase of the oxidation time results in a decrease in the diameter of the Fe core and an increase in shell thickness. Figure 2b shows the results after 20 minutes of Fe_3_O_4_ in the face-centered cubic (*fcc*) phase and Fe in the *bcc* phase. After 24 hours, oxidation is complete and only a polycrystalline Fe_3_O_4_ structure is observed.

**Figure 3:**
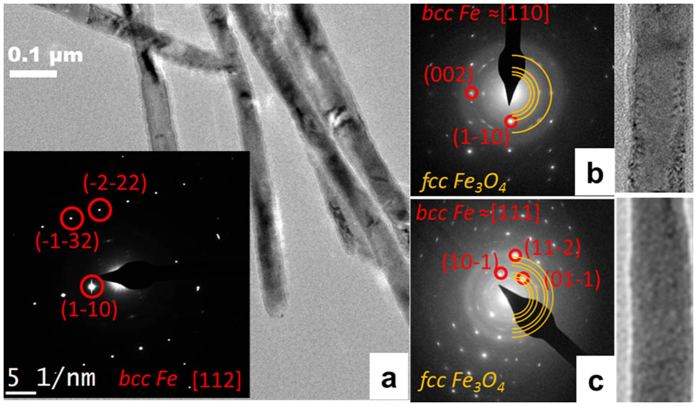
(**a**) Bright field TEM image and SAED pattern of single-crystalline Fe NWs, Fe-Fe_3_O_4_ core-shell NWs (**b**) after 1 hour of annealing and (**c**) after 72 hours of annealing.

Figure 3a shows a TEM image and SAED pattern of single-crystal Fe NWs. The crystallinity is pronouncedly different from those of the ones of the polycrystalline NWs shown in Fig. 2a. Single micrometer *bcc*-structured crystals are clearly visible and the single-crystal phase is confirmed by SAED. Note that due to the specific growth conditions of Fe in the AOT, the first section of the NWs, which is attached to the Au electrode, exhibits a polycrystalline structure. This polycrystalline area is typically a few hundred nanometers long and corresponds to the penetration depth of the Au into the pores of the AOT during sputtering of the Au electrode. After annealing, rings from the *fcc* Fe_3_O_4_ shell appear in the SAED pattern (Fig. 3b). In contrast to the polycrystalline NWs, the single-crystalline NWs do not fully oxidize. Shell thickness was 3–5 nm after 10 minutes of annealing, 5–7 nm after 1 hour of annealing and 10–12 nm after 24 hours, which was the maximum shell thickness we could obtain (no further thickness was obtained even after 72 hours) (Fig. 3c).

**Figure 4:**
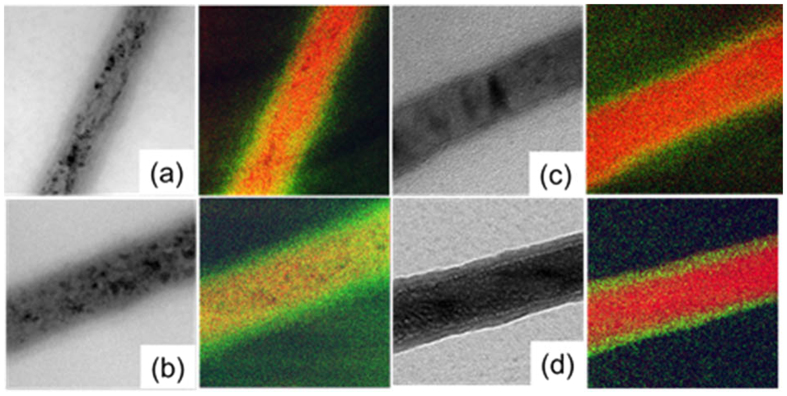
Bright field and color-coded EF-TEM images of the Fe L_23_ edge (red) and O K edge (green) of polycrystalline Fe NWs after (**a**) 20 min and (**b**) 24 hours annealing and single crystalline Fe NWs after (**c**) 1 hour and (**d**) 72 hours annealing.

The thicknesses of the cores and shells after different oxidation times were measured by energy-filtered TEM (EF-TEM). Figure 4 shows typical EF-TEM images of polycrystalline and single-crystalline Fe NWs, which were obtained by combining the Fe L_23_ edge and O K edge. In core-shell NWs, the O clearly concentrates at the edges (Fig. 4a,c,d), while in fully oxidized NWs, O is distributed across the entire volume (Fig. 4b).

**Figure 5:**
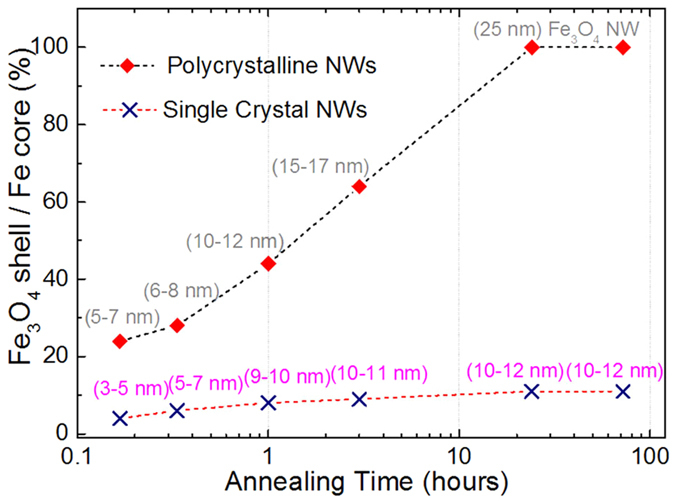
Shell to core ratio for different annealing times of polycrystalline and single-crystal NWs, showing that after 24 hours, the polycrystalline NWs are fully oxidized, while the maximum shell thickness for single-crystal NWs is 12 nm, even after 72 hours.

Shell thicknesses following annealing times are summarized in Fig. 5. Shell thickness of polycrystalline NWs increases linearly with annealing time until pure Fe_3_O_4_ NWs are obtained after 24 hours (Fig. 4b). Single-crystal NWs oxidize slower such that shell thickness saturates at around 12 nm (Fig. 4d). This can be attributed to the absence of grain boundaries that are required for heat-assisted oxygen diffusion. The ability of single-crystal core-shell NWs to resist complete oxidation is a great advantage for applications that require high-temperature operations, while they maintain the magnetic properties of the Fe core.

**Figure 6:**
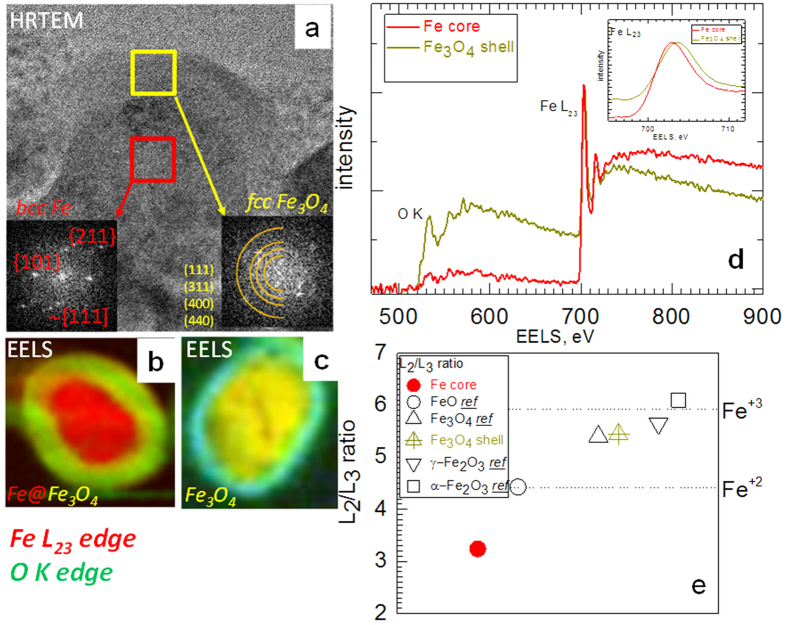
(**a**) HR-TEM cross-sectional image of a single-crystal core-shell Fe nanowire (after 72 hours of annealing of a single-crystal Fe nanowire). EELS spectra of the cross-section of an Fe nanowire annealed for 24 hours: (**b**) the single-crystalline portion and (**c**) the polycrystalline portion (the end of the nanowire, where the growth started from). (**d**) A spectrum obtained by EELS from core and shell regions. (**e**) White-line ratios of the Fe L_23_ edge for the core, shell and reference samples of different iron oxides measured using the same techniques.

Figure 6a shows a high-resolution TEM (HRTEM) cross-sectional image of a single-crystal core-shell Fe nanowire after 72 hours of annealing. Agreement of the SAED patterns with those of the reference Fe_3_O_4_ does not necessarily indicate that the phase of the oxide shell corresponds to magnetite. This is because the electron diffraction ring patterns of Fe_3_O_4_ and γ-Fe_2_O_3_ are very similar^36^. Thus, the oxide shell can be either Fe_3_O_4_ or γ-Fe_2_O_3_ or a mixture of both. Spectra obtained by electron energy loss spectroscopy (EELS) for a specific atomic species are influenced by both the coordination chemistry and the valence state of the atomic species being measured^37^. Figure 6b shows the EELS map of the cross-section of a core-shell NW (single-crystalline Fe NW after 24 hours of annealing) prepared by focused ion beam protocol. Two distinguished regions are clearly visible namely, the Fe core and the surrounding Fe-O shell. In contrast, the EELS map of the cross-section of a polycrystalline section (at the end of the NW, where the growth started from) shown in Fig. 6c denotes a completely oxidized Fe-O state. The blue circle around the NW arises from the Cr edge, caused by remnants of the chrome solution used to release the NWs from the AOT (see the Methods section).

Depending on the phase and the valence of Fe in the Fe-O structure, several Fe-O configurations are possible (see the Supplementary Information)^36^. The EELS fine structure of both the O K-edge and the Fe L_23_-edges imprints these structural differences and therefore can be used to identify a specific Fe-oxide phase^36^,^37^. The valence state of Fe can be determined from the chemical shift (dependence of the edge position with respect to the valence), fine structural features (splitting of the peaks), and the white-line ratios of the Fe L_2_ and Fe L_3_ spectra^38^.

Figure 6d shows the EELS spectra recorded from the Fe core and Fe-O shell. The chemical shift of the Fe edge is clearly observable from the inset. To determine the valence state of the shell, we calculated the white-line ratios of the Fe L_2_ and Fe L_3_ spectra and compared them with those measured from the reference samples (for details see Supplementary Materials). Figure 6e illustrates that the value of the white-line ratio from the NW shell is in the region that corresponds to the magnetite oxidation level^37^,^39^,^40^.

**Figure 7:**
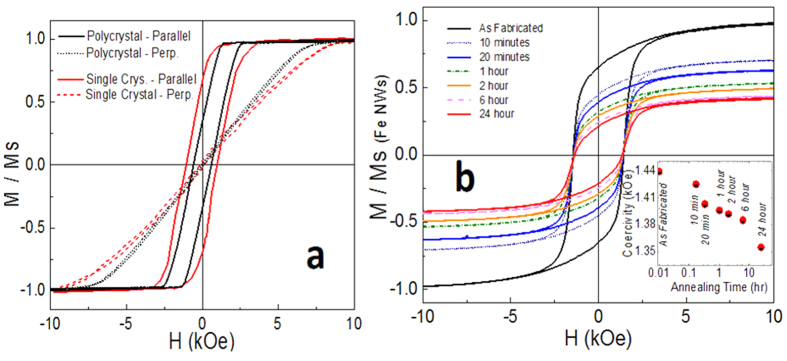
Magnetization of nanowires (NWs) as a function of the applied field measured with a vibrating sample magnetometer. (**a**) Polycrystalline and single-crystal Fe NWs in aluminum oxide templates with the field applied parallel and perpendicular to the NWs. (**b**) Magnetization curves of polycrystalline Fe NWs and core-shell NWs with 24 hours of annealing time released from the aluminum oxide templates and dispersed on a silicon substrate. The field was applied in plane with the substrate. In inset: The coercivity dependence.

Magnetization curves were obtained using a vibrating sample magnetometer (VSM). The results of Fe NWs inside AOTs (Fig. 7a) confirm a longitudinal magnetic anisotropy (with magnetization easy axis parallel to the long axes of NWs), which can be related to the strong shape anisotropy of the high aspect ratio NWs^22^. A higher remanent magnetization is observed for single-crystal Fe NWs than for polycrystalline NWs, which is ascribed to a higher term of magnetocrystalline anisotropy^22^.

The magnetization curves of polycrystalline Fe NWs released from the AOTs and dispersed on a silicon substrate show a coercivity of around 1400 Oe (Fig. 7b), which is nearly double that of the NWs inside the AOT, which reach just 675 Oe. This can be at least partially attributed to the effect of the misalignment of the NWs with respect to the field. Inside the AOT, the NWs are parallel to the direction of the external magnetic field. NWs dispersed on the substrate have a random orientation compared to the NWs in AOTs, and the switching field increases with increasing angle between the axis of the NWs and the direction of the applied field^24^.

The hysteresis loops of core-shell NWs (Fig. 7b) differ considerably from those of Fe NWs, namely, they showed a lower saturation magnetization, a lower remanence, and a smaller coercive field. Reduced saturation magnetization is caused by the smaller magnetic moment of Fe_3_O_4_ compared to Fe^41^,^42^. This also contributes to a slight reduction of coercivity (see inset of Fig. 7b) that strongly depends on the shape anisotropy value of the NWs, which is proportional to the NW magnetization^22^.

**Figure 8:**
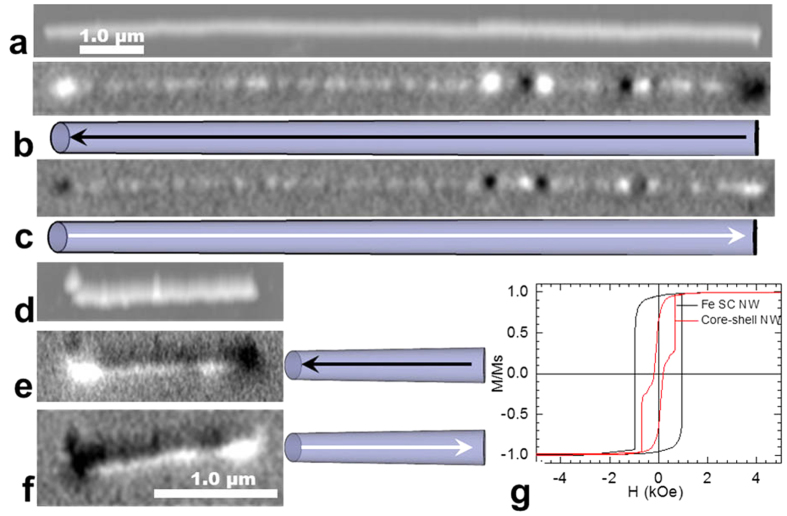
Topography (**a**,**d**) magnetic force microscopy (MFM) images at remanence of (**a–c**) a polycrystalline Fe nanowire (NW) and (**d–f**) a single-crystalline Fe NW; (**b**,**e**,**c**,**f**) MFM images at remanence after saturation in the opposite direction. The drawings schematically show the direction of NW magnetization. (**g**) Micromagnetic simulation results of magnetic loops of single-crystalline (SC) Fe NWs before and after 1-hour annealing (core-shell NW). The saturation magnetization values of the Fe core (2.01 T) and magnetite shell (0.28 T) used in the simulations were extracted from electron holography studies.

To investigate the magnetic properties of individual core-shell NWs, magnetic force microscopy (MFM) studies under variable fields have been conducted. A NW was first saturated under a field applied parallel to its axis and then scanned at remanence. Afterwards, the applied field was reversed in steps of 20 Oe. After each step, the field was removed to avoid any effects caused by the stray field from the magnetic tip before conducting a scan. The coercivity was defined as the field at which the MFM contrast of the NW was reversed. At remanence, the black and white points at the ends of the NW were caused by strong magnetic stray fields, confirming the single-domain magnetic state with the magnetization along the axis of the NW for both polycrystalline (Fig. 8a–c) and single-crystal (Fig. 8d–f) NWs. The contrasts found in MFM along the polycrystalline NW are due to stray fields originating from local defects in the shape of the NW. After saturating the NWs in the opposite direction, the contrast in MFM at remanence appears reversed, indicating a change in the direction of magnetization. Magnetization reversal from one saturation state to the opposite state occurs at the coercive fields of approximately 695 Oe for polycrystalline and 1250 Oe for single-crystalline NWs, which is slightly higher than the coercivity of the array (Fig. 7a). This magnetization reversal mechanism is in agreement with previously reported micromagnetic simulations, showing a reversal process by switching the direction of magnetization at the coercive field via propagation of the vortex domain wall^22^.

**Figure 9:**
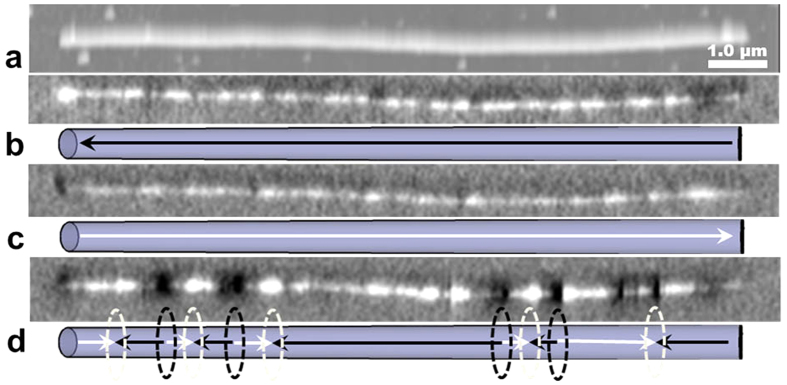
Topography (**a**) and MFM images (**b–d**) of a polycrystalline core-shell nanowire (20 min annealing). (**b**,**c**) at remanence after saturation in opposite directions parallel to the length of the nanowire. (**d**) Multi-domain state at 1.05 kOe external magnetic field. The schematics in (**b–d**) show the magnetization direction (arrows) and domain wall positions (ellipses).

The core-shell geometry significantly modifies the magnetic behavior of individual NWs. Figures 9b-9c shows MFM images at the remanent states of a core-shell NW (polycrystalline Fe NW annealed for 20 min) after magnetic saturation with a field parallel to the long axis of the NW as indicated in the schematics view. Similar to Fe NWs without a shell, single-domain magnetic states are observed over a wide field range. Magnetization switching occurs between 870 and 1100 Oe, where the magnetization reversal process includes multi-domain states (Fig. 9d). It is reasonable to assume that this is caused by strong pinning sites introduced by the core-shell structure. The higher coercivity compared to Fe NWs without a shell can be explained by the significant change in diameter (of the Fe core) due to oxidation^22^. The multi-domain structure is maintained after removing the field, resulting in different remanence values. This unique feature allows “programming” of different magnetization values into NWs, which could prove very useful for various applications.

In contrast to core-shell NWs produced by annealing polycrystalline Fe NWs, those produced from single-crystal Fe NWs did not form multi-domain states. Both single-crystal Fe NWs and core-shell NWs were in a single-domain state and magnetization switching occurred at the coercive field of 1250 Oe for Fe NWs and 540 Oe for core-shell NWs. This reduction in coercivity can be attributed to the change in the length of the Fe core (from 1.5 μm to 1.1 μm), due to the oxidation of the polycrystalline portion at the end of the NW, which affects the shape anisotropy more significantly than the change of the core’s diameter. These findings were confirmed by finite element micromagnetic simulations (Fig. 8g).

**Figure 10:**
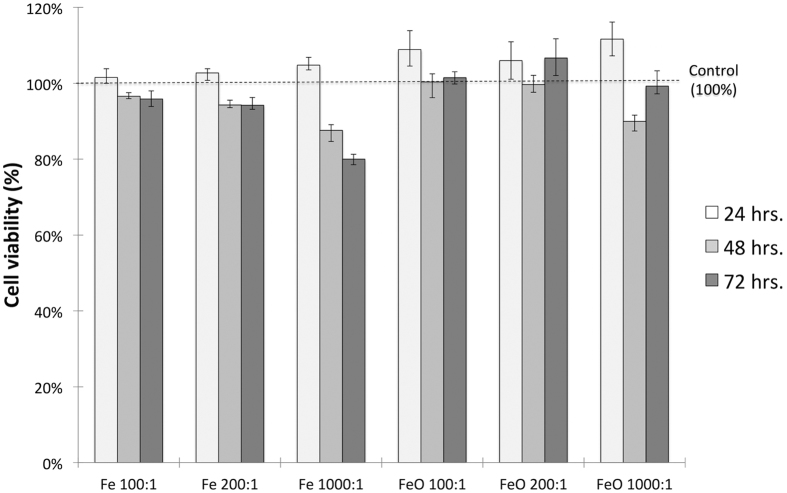
An MTT assay for the assessment of the viability of HCT 116 cells incubated with Fe and Fe-Fe_3_O_4_ core-shell NWs for 24, 48, and 72 hours. The concentrations on the x-axis denote the NW-to-cell ratio. The data represent mean ± range, and the number of replicas was n = 3.

The biocompatibility of the Fe and Fe-Fe_3_O_4_ core-shell NWs was assessed using the MTT assay, which measures the metabolic activity of the cell and is therefore a good indicator of cell health and viability (see Methods). Figure 10 shows the cell viability of HCT 116 cells incubated with three concentrations of Fe and Fe-Fe_3_O_4_ NWs for 24, 48, and 72 hours. Core-shell NWs appear to be more biocompatible than Fe NWs. It is possible that this effect is due to the prevention of intracellular dissolution of the NWs, due to the presence of the passivation Fe_3_O_4_ shell, as has been suggested elsewhere^43^,^44^. There was no statistically significant decrease in cell viability for most of the conditions tested for the two NW materials. We only observed a significant drop in cell viability at the highest concentrations and incubation times of Fe NWs. In general, these results show that both Fe and the core-shell NWs possess a good biocompatibility and are not cytotoxic to the model cell line used.

## Discussion

We have demonstrated a simple and effective preparation method for Fe-Fe_3_O_4_ core-shell NWs in which the thickness of the shell and the diameter of the core are tunable. This enables tailoring of the magnetic properties, in particular the values of saturation and remanence magnetizations, within a wide range. TEM studies of the shell thicknesses and morphology/structure of the materials confirmed that these core-shell NWs can be synthesized with either polycrystalline or single-crystal Fe cores and distinctly different characteristics. In all cases, a magnetite shell was formed. The oxide shell of polycrystalline Fe NWs grows continuously with annealing time until the disappearance of the core. However, the thickness of the oxide shell of single-crystal Fe NWs does not increase beyond about 10 nm, and the Fe core is maintained. This is specifically attractive for application to harsh environments, where such core-shell NWs can provide the magnetic properties of the Fe core at high temperatures. Magnetization measurements revealed that the saturation and remanent magnetizations depend on the oxide shell thickness, i.e. they decrease with increasing shell thickness. Meanwhile, the coercive field value reflects the competition between the effect of the magnetite shell (it reduces coercivity) and the size of the iron core’s diameter (a smaller diameter increases coercivity) and changes only slightly. Our method presents a simple technique for tailoring the magnetic properties of the NWs. On one hand, NWs with a thicker shell have a smaller remanence, which minimizes magnetostatic interactions between NWs and contributes to their dispersion in solutions. On the other hand, NWs with a thinner shell maintain a higher remanence and can be used as nano-sized permanent magnets. In-between, the NWs offer many opportunities for applications in cell separation, corrosion monitoring etc. In addition, polycrystalline core-shell NWs showed several remanent magnetization states, a unique feature, which could be exploited in the future to program different magnetization values into the same NWs.

## Methods

### NW fabrication

Fe NWs were fabricated by a well-known process using electrodeposition into self-ordered AOT synthesized by a two-step anodization process using oxalic acid (see Supplementary Information for details).

### Formation of the Fe-Fe_3_O_4_ core-shell

Fe-Fe_3_O_4_ core-shell structures were formed by oxidizing the Fe NWs in an oven at 150 °C for 10 minutes to 72 hours. To this end, the electrodeposited NWs were released from the AOT using chemical etching (Cr_2_O_3_/H_3_PO_4_*H_2_O solution at 40 °C).

### Characterization

Electron microscopy studies were performed on a Cs corrected (scanning) TEM Titan 60–300 (FEI, Netherlands), operated at 300 kV. To study the crystal structure of individual NWs, the AOT membranes were dissolved and the NWs were dispersed in ethanol. Cross sections of core-shell NWs dispersed on a Si substrate were prepared by the conventional focus ion beam protocol. The EELS and EF-TEM experiments were performed with a post-column high-resolution Gatan energy filtering spectrometer. The optical conditions of the microscope for EELS imaging and spectroscopy were defined to obtain a probe-size of 0.2 nm, with a convergence semi-angle of 10 mrad, and a 12 mrad collection semi-angle.

The magnetic properties of the NW arrays were studied using a VSM. The magnetization curves were measured under magnetic fields up to 10 kOe with the field applied parallel and perpendicular to the axes of the NWs.

MFM images were recorded in lift-off mode (100-nm distance) with an MFP-3D-Bio from an Asylum Research scanning probe microscope using standard atomic force microscopy nano-sensor probes with a magnetic coating. A single NW was selected using scanning electron microscopy and its position was marked with a focused ion beam. MFM measurements were done at the remanent state after applying magnetic fields parallel to the axes of the NWs using a variable magnetic field module (±5kOe).

The demagnetization process of NWs was simulated by the MagPar package with finite element discretization^45^. The average finite element discretization size was chosen to be 2 nm. The magnetic parameters of Fe were taken from Ref. 22. The magnetization of magnetite was extracted from electron holography measurements −0.28 T.

### Cytotoxicity assessment

The MTT (3-(4, 5-dimethylthiazol-2yl)-2, 5-diphenyl tetrazolium bromide) assay was used to assess the cytotoxicity of the Fe and core-shell NWs. HCT 116 colon carcinoma epithelial cells (ATCC® CCL247TM) were cultured in McCoy’s 5A modified medium (Gibco®), supplemented with 10% fetal bovine serum (Gibco®) and L-glutamine and grown in a 37 °C humidified incubator with 5% CO_2_. Upon reaching 80% confluence, cells were detached from the culture flasks with 0.25% trypsin-EDTA and counted using trypan blue staining. The cells were cultured in 96-well plates, and after 24 hours of stabilization, they were treated with NWs at different NW-to-cell ratios (number of NWs per cell in the culture at the time of treatment). After the desired incubation times, the cell medium was discarded and replaced with 10% MTT solution—5 mg/mL in phosphate buffered saline—in McCoy’s medium. The cells were incubated for two hours, and then the medium was discarded and replaced with 90% dimethyl sulfoxide—10% sodium dodecyl sulfate lysis buffer to dissolve the MTT reduction products. Cell viability was evaluated through optical density with a microplate reader (XMarkTM, Bio-Rad) using a wavelength of 570 nm and a background wavelength of 630 nm.

## Additional Information

**How to cite this article**: Ivanov, Y. P. *et al.* Tunable magnetic nanowires for biomedical and harsh environment applications. *Sci. Rep.* **6**, 24189; doi: 10.1038/srep24189 (2016).

## Supplementary Material

Supplementary Information
