# Supplementary material for: Tunable magnetic nanowires for biomedical and harsh environment applications
Source: Sci Rep. 2016 Apr 13;6:24189. doi: 10.1038/srep24189 (PMC4829833; doi:10.1038/srep24189)
Supplement: Supplementary Information [file srep24189-s1.doc]

Supplementary Information

**Tunable magnetic nanowires for biomedical and harsh environment applications**

Yurii P. Ivanov^1+^, Ahmed Alfadhel^1+^, Mohammed Alnassar^1^, Jose E. Perez^2^, Manuel Vázquez^3^, Andrey Chuvilin^4,5^, Jürgen Kosel^1^*

^1^Computer, Electrical and Mathematical Sciences and Engineering Division (CEMSE), King Abdullah University of Science and Technology (KAUST), Thuwal, 23955, Saudi Arabia

^2^Biological and Environmental Sciences and Engineering Division (BESE), King Abdullah University of Science and Technology (KAUST), Thuwal, 23955, Saudi Arabia

^3^Institute of Materials Science of Madrid, CSIC, 28049 Madrid, Spain

^4^CIC nanoGUNE Consolider, Av. de Tolosa 76, 20018, San Sebastian, Spain

^5^IKERBASQUE, Basque Foundation for Science, Maria Diaz de Haro 3, 48013, Bilbao, Spain

***Email: [jurgen.kosel@kaust.edu.sa](mailto:ahmed.fadhel@kaust.edu.sa)

^+^ These authors contributed equally to this work.

Fe-O system details.

For the Fe-O system, depending on the phase and the valence of Fe in the structure, several types of Fe-O coordination configurations are possible. Fe-O has the rock salt structure with space group Fm3m (225) and a lattice constant of 4.3088 Å. The Fe has a valence of +2 and is ideally coordinated by six O atoms (octahedral configuration). Fe_3_O_4_ has the inverse spinel structure with space group Fd3m (227) and a lattice constant of 8.3941 Å. The Fe in Fe_3_O_4_ has a mixed valence of Fe^2+^ and Fe^3+^ with a ratio of Fe^2+^/Fe^3+^ = 1/2. The Fe occupies both tetrahedral and octahedral sites. γ-Fe_2_O_3_ has cubic symmetry with space group P4332 (212) and a lattice constant of 8.3474 Å. The Fe has a valence of +3. α-Fe_2_O_3_ has a corundum-type structure with the space group R3c (167) and lattice constants of a=5.03521 Å and c=13.7508 Å. Fe^3+^ occupies two-thirds of the octahedral sites confined by the nearly ideal hexagonal close-packed O lattice.

Details of the Fe nanowire fabrication.

The AOTs contained uniform and hexagonally arranged nanopores, as confirmed by scanning electron microscopy imaging. Prior to anodization, high-purity (99.999%) aluminum disks were cleaned in an acetone bath using ultrasound, and electropolished with mixture of perchloric acid and ethanol (HClO_4_:C_2_H_5_OH = 1:4). The first anodization was performed using an oxalic acid solution as electrolyte at 2 °C and an anodization voltage of 40 V for 24 hours. Then, the sample was immersed in chromsolution (mixture of Chromium (VI) Oxide and Phosphoric acid) at 40 ^o^C for 12 hours to dissolve the alumina. The second anodization was performed for 20 hours to form a 40 μm thick alumina membrane with highly ordered pores. The non-oxidized aluminum at the back side was etched away using a copper solution (mixture of Copper(II) chloride and Hydrochloric acid) and then the alumina barrier layer was removed using phosphoric acid (10 wt% H_3_PO_4_), leaving the pores open from both sides. A 5 nm thick gold electrode was sputter deposited onto one side of the porous membrane to provide a conducting layer for the electrodeposition process, and which was etched away by reactive ion etching after the deposition of the NWs. Fe was then DC electrodeposited from an aqueous electrolyte. Polycrystalline Fe NWs were grown at room temperature in an aqueous solution of 45 g/L FeSO_4_, 30 g/L Boric acid (H_3_BO_3_) and 1 g/L Ascorbic acid (C_6_H_8_O_6_). Single crystalline Fe NWs were grown in an aqueous bath with a solution of 5.4 g/L Iron (II) Sulfate Heptahydrate (FeSO_4_·7H_2_O), and 10.3 g/L Sodium Citrate Dihydrate (HOC(COONa)(CH_2_ COONa)_2_·2H_2_O). Electrodeposition was carried out at room temperature, under constant stirring and at a bias voltage of –1.1V. The resulting polycrystalline NWs were 15 μm long and 50 nm in diameter, and the single crystalline NWs were 1.5 μm long and 50 nm in diameter.

EELS spectra from reference samples with different iron oxides.


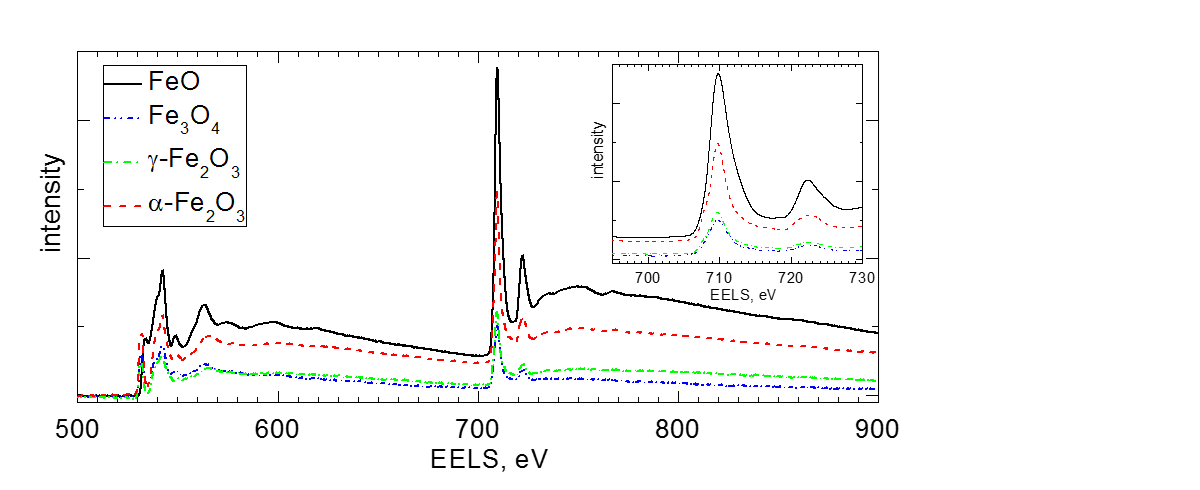


**Supplementary Figure S1.** EELS spectra of the reference samples of different iron oxides.

Nanowire release and number calculation for biocompatibility study.

Fe NWs were released using a 1M NaOH solution. Briefly, the NWs were put in an ultrasonic bath for two minutes, and then the NaOH solution was replaced using a magnet to avoid the loss of NWs. This process was repeated until the dispersion of the NWs was homogeneous, at which point they were washed thoroughly with ethanol.

To estimate the number of NWs, the nanopores were individually counted using a scanning electron microscope image of the top-view of a defined area. As each nanopore equals one NW, the total number counted was rescaled to the total deposition area. The ImageJ software was used for this purpose.
